# Dementia at the End of Life and Family Partners: A Symbolic Interactionist Perspective on Communication

**DOI:** 10.3390/bs7030042

**Published:** 2017-07-09

**Authors:** Christopher Johnson, Jordan Kelch, Roxanna Johnson

**Affiliations:** 1 Department of Sociology, 601 University Drive, Texas State University, San Marcos, TX 78666-4684, USA; j_k92@txstate.edu; 2 Gerontologist and Dementia Specialist, Aging Consultants, Austin, TX 78733, USA; agingconsultants@gmail.com

**Keywords:** communication, symbolic interaction, end of life, family care partners, persons living with dementia

## Abstract

People with dementia are not dying; they are experiencing changes in the brain. This paper utilizes a symbolic interaction theoretical perspective to outline communicative alternatives to polypharmacy. There is a growing interest in sociological interventions to untangle the “disordered discourses” associated with dementia. Such practices challenge common stigmas attached to dementia as an “ongoing funeral” or “death certificate.” Changing the expectations, attitudes and communication patterns of family care partners can positively impact them and the person living with dementia at the end of life. This paper delineates multiple non-verbal communication interventions (e.g., the trip back in time, dementia citizenship and sensory engagement modalities) to explore techniques to engage persons with advanced dementia.

## 1. Introduction

This article uses a symbolic interactionist perspective to view the salience of developing ways to communicate with end of life persons living with dementia. This paper is about radical social change in care partnering with the “persons living with dementia” (PLWD). Elements of symbolic interactionist theory provide an explanation of experiences and communication patterns of PLWD and their significant others at the end of life [1,2]. The phrase “end of life” is used as a relative term because the dying process begins at birth. PLWD are not dying from their diseases, they are living with them. Moreover, they are aging like all of us; some are aging more rapidly than others. This paper disrupts the commonly held assumptions about end of life dementia care. The authors further inspect partnerships and offer new therapeutic symbols to surround such relationships. When you enter the social world of a PLWD at the end of life, there are already well formed negative public perceptions driven by sociological forces of stigma and ageism [3]. This paper focuses on social exclusion and loss of dementia citizenship and offers strategies to alter family interactions and communication styles to more effectively connect with PLWD at the end of life. The symbolic interactions suggested are non-normative and offer methods of making social connections at the end of life with PLWD with aphasia. All PLWD have disabilities with unexplored abilities and it is those unexplored strengths that we seek to identify for families. The medical definition of dementia views dementia as loss. The Alzheimer’s Association website defines dementia as a general term for the loss of memory and other mental abilities severe enough to interfere with daily life [4]. However, this paper follows the experiential school of thought which defines dementia as a “shift in the way we experience the world” [5].

## 2. Family Perceptions of End of Life Dementia as Tragedy and Loss

Sociologist W.I. Thomas developed a theory of sociology which maintains, “If men define situations as real, they are real in their consequences” [6] (p. 62). In the context of the disease model of dementia, the subjective interpretation of being diagnosed with the label of dementia influences actions on both the part of the “patient” and the family. In partnerships, all participants negotiate shared meanings. Family behaviors are affected by negative views of dementia that already exist in American culture. Drawing on the work of W.I. Thomas, Sociologist Robert Merton maintains that any definition of a situation influences the present [7]. The negative expectations and behaviors of loved ones do adversely affect PLWD. Societal views which associate aging with memory loss are common and such stigmas affect the perceptions of family members [3]. It is possible for negative stigmas to be projected onto the PLWD by significant others [8]. 

Dementia as a tragedy motif has a long history in American medicine. Gerontologists today attack traditional symbolic representations of dementia as loss [9]. Some scholars point out that symbolic meanings attached to the label dementia are interpreted, embodied, or resisted by families in their social contexts [10]. These processes are shaped according to their social location (ethnicity, gender, and social class) and each PLWD’s social history. The PLWD’s “death certificate” is not signed with the label dementia. The tragedy lies in the social consequences of stigmas attached to diagnostic labels, described in the United Kingdom mainstream press as a “panic blame discourse” producing views of dementia as inevitable loss and decline while simultaneously telling stories about ways of staving off the disease [10]. This reframes dementia as something within the realm of individual choices and suggests a potential judgment of PLWD as not having aged successfully. Gerontological literature over the years has been full of negative labels for dementia associating it with dying. Decades ago, social workers described the grieving process of families who live with a PLWD as experiencing an “ongoing funeral” [11]. As a result, finding a positive or hopeful view of the disability of dementia amidst such “malignant” metaphors are virtually impossible [12].

A physician study discusses the lack of reciprocity in family members caring for PLWD in end of life conditions [13]. Physicians can offer more positive views of dementia as a disability with abilities. Doctors are in a position to dispel myths, stereotypes and stigmas of dementia with family members. The emphasis is on the potential that medical professionals have to educate care partners of PLWD by providing information on better non-verbal communication techniques at the end of life [13]. Implementing these skills would enhance PLWD’s ability to reciprocate. For example, “Such changes have implications for improved care and quality of life through the continued maintenance of social inclusion and perceptions of personhood” [13] (p. 1). Doctors can take the lead in destigmatizing PLWD as persons with disabilities rather than persons who are dying or dead. Studies focusing on dementia as a tragedy theme are prolific and point out different ways in which dementia is experienced negatively. They also suggest that the cultural meanings attached to dementia vary by society [14]. Sociological research in India delineates cultural representations of dementia as being mixed with other symbolic meanings such as the social expectations of aging [15]. By understanding the different symbols attached to dementia that are culturally-based, families can identify how particular meanings associated with dementia govern others’ behaviors and are sociologically significant [16]. This perspective allows families to see the implications of cultural representations for those living with dementia, and how these implications may differ according to a person’s socio-cultural background. 

Industrialized societies offer a proliferation of scary dementia stories and labels that are reflected in mainstream media, fictional books and literature. Accounts of dementia present this condition as a kind of living death for its sufferers and to their family members [17]. Some have argued that both the bio-medicalization of dementia and the social construction of PLWD as “zombies” create fear of the disease [18]. Likewise, this fear marginalizes and disempowers a person given the label of dementia [16]. Public fear translates into “dehumanization based on disgust and terror and influences perceptions of family members, including children” [18] (p. 70). Such symbolic representations create public reactions of revulsion and fear of losing your mind that people associate with aging. The language of loss and determinism pervades these cultural symbols of ageism and stigma. For example, the rising tide of dementia has been described as a “tsunami”, suggesting an unstoppable wave [19]. As a result, these stigmas lay the ground work for negative perceptions for families of persons with dementia [18]. Kitwood [12] referred to a “malignant social psychology” of dementia, where families experience unfair discrimination, disempowerment and prejudices (stigma, stereotyping etc.) directed toward their loved one with dementia [12]. Others have used such examples as the foundation for his theory of “malignant social positioning” [20]. Labels of PLWD as “dying” or being in a “vegetative state” serve as triggers for the frequent social disengagement of family members, especially at the end of life.

## 3. End of Life and the ‘Trip Back in Time’

**Figure 1:**
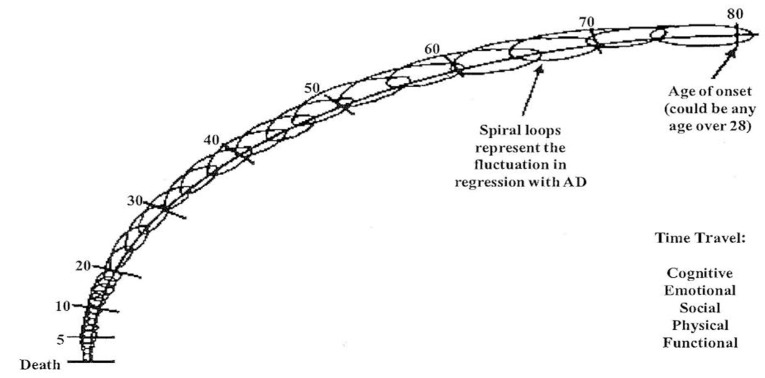
Alzheimer’s disease (AD) as a “trip back in time”.

Johnson and Johnson’s concept of the ‘trip back in time’ offers a paradigm which explains how persons experience time travel through the cognitive, emotional, social, physical and functional domains with Alzheimer’s disease (AD) [9]. The ‘trip back in time’ utilizes a downward spiral diagram with connecting loops (see Figure 1) to demonstrate the fluctuating, non-linear, but progressive course of the disability. The ‘trip back in time’ from age of onset back to early childhood is both fluid and fluctuating for the AD person. The ‘trip back in time’ model can account for the person’s ability to fluctuate in both memory and recognition of family members as they travel back through time. The capabilities of the AD persons vary throughout, beginning with the changes in short term memory followed by long term memory. The AD person’s physical time travel traverses from normal to super human strength, to reduced ambulation, and finally to the fetal position (i.e., bedridden) similar to a baby in the womb. The connecting loops progressing downward also account for adult development in reverse as identified by previous research [21]. Additionally, the ‘trip back in time’ model allows for the non-linear variances on a daily basis through time travel in all domains. The connected loops demonstrate how an AD person can make small or quantum leaps springing up from the past to the present for brief periods of time. Past studies have suggested theoretical time travel although it had not been identified as a ‘trip back in time’ to infancy or what is referred to here as end of life care [9]. Ironically, both the bedridden AD person who has time traveled back to infancy and the infant are more in touch with their emotions than any other time in their life. This is a time when emotions are raw with no pretenses. It is healthy to be in touch with emotions although it can make families uncomfortable, especially when PLWD express sadness, loneliness or pain by crying.

Case Study, Susan and Dan: An 80-year-old woman named Susan diagnosed with AD experiences a downward spiral back and forth through time, traveling and revisiting persons, pleasant and traumatic events that occurred throughout her life. She travels in her mind back to age 20 and no longer recognizes significant others who are currently in her life but does recognize pictures of her parents and siblings. Imagine that Susan is visited by her nuclear family and grandchildren but she mislabels or mixes up the generations calling her son by her husband’s name. The family is frustrated by these actions and assumes Susan is confused or “crazy.”

Again, Susan has traveled back to age 20 in her mind and upon looking in the mirror sees an 80-year-old face and asks “What are you doing in my bathroom?” All along it was the same person, Susan, with cognitive fluctuations. Later, Susan’s 84-year-old husband Dan enters her room and identifies himself as her husband, although in her mind she is 20 years old. When Dan identified himself as her husband he imposed his “reality” (time frame and definition of the self) upon Susan. This is extremely confusing for Susan and can trigger aggression and fear of Dan. Validation of the AD person’s experiences is empowering for them because to them the experiences are real [22]. Therefore, care partners are trained on how to roughly identify where their loved one with AD is in the time travel experience [22]. Then, they validate the AD person’s definition of the situation and self. Validations are accomplished through various forms of non-verbal communication such as sharing old pictures of family and friends from yesteryear in photo albums, listening to music from the person’s distant past and other special interests. Instead of labeling the person as having delusions, care partners are trained to recognize the ‘trip back in time’ process, no longer invalidating their reality [22]. Understanding the ‘trip back in time’ model and how to join one on their time travel journey with appropriate communication can help avoid the frustration and heartache from an unnecessary reality orientation. Families who understand this model refrain or resist identifying current grandchildren and nuclear family members. These care partners will instead join the person on the trip back in time to connect in their time frame.

## 4. Symbolic Interactions with End of Life PLWD

For family care partners who are in daily interaction with PLWD, the symbolic label “dementia” triggers grief due to many social factors. Such disease labels cause a great deal of frustration and pain for families as they attempt to deconstruct their view of the self of the PLWD. Families grieve the person (self) they once knew and then try to reconstruct a new “dementia as tragedy” view of the PLWD in what symbolic interactionists call the “sick role.” Family usually see the loved one as a “victim of a disease” rather than a person with a disability. This can be the case when there is lack family dementia education in understanding time travel and a more holistic view of the disability through the disease process. 

There is a great deal of ambiguity around how social expectations drive care partners’ behaviors. Loved ones wonder what kind of roles should be enacted, what to say and what to do around PLWD in the new status of being bedridden. Significant others either adjust their behaviors to accommodate PLWD or they choose to disengage, usually with justifications for doing so. Communications and interactions between PLWD, professionals and family care partners construct a perceptual framework for what needs to be done. Culture and society play a role in this construction as well. Some of this framework is based upon the preconceived views of significant others (family and friends) about PLWD’s bedridden status and her inability to verbally communicate. This perception of “an ongoing funeral” for PLWD is socially negotiated and constructed, aided by stereotypes and the media. The meanings constructed of the life of the bedridden persons are created by symbolic interactions that are shared between the PLWD, staff and significant others within the culture in which they live. It is through dementia education that care partners can learn how to effectively join the end of life PLWD on their ‘trip back in time.’

## 5. Medicalized Care and End of Life Dementia Care

Bedridden PLWD who are in distress in institutions are often sedated with medications. However, family care partners who understand the role of medications at the end of life dementia care can be advocates for sociological interventions when appropriate. An astounding statistic is that 13% of the population comprises elders over 65 years of age, yet they consume 34% of prescription drugs [23]. According to some, the pharmaceutical industry is driven by profit and most other concerns are usually secondary [24]. Elders are the largest consumers of prescription medications [25]. End of life dementia care has been increasingly medicalized over the course of the past few decades. Some medications are necessary when caring for PLWD due to frequent comorbid health conditions [26]. Polypharmacy or over use of medications can be excessive and it does impact social engagement between the family care partners and the PLWD. There is a plethora of new research on sociological interventions for end of life PLWD. The sociological (non-pharmaceutical) interventions for end of life care can be overlooked by an emphasis on medical solutions to distress in PLWD [27]. Recent literature suggests that quality of life for PLWD can be significantly improved when the pharmacological treatment is sufficient but not excessive [27]. Anti-psychotic medications are often utilized as both treatment and chemical restraint when the care plan does not expressly limit their use [28,29]. 

Pain or discomfort is frequently difficult for PLWD to communicate, especially if they are deemed non-communicative [30]. As a result, family care partners in long- term care environments may stop making efforts that directly affect medications and dosages. Research suggests that under and overmedication as a result of poor communication can increase cognitive impairments in PLWD [31]. A recent study interviewed doctors and disclosed that anti-psychotic medications were frequently prescribed based on the PLWD’s exhibition of aggression or anger [32]. For the bedridden PLWD, behavioral symptoms can accompany chronic boredom or loneliness due to sociological factors or lack of social engagement and meaningful activities [33].

## 6. Sensory Engagement Modalities for End of Life Person Centered Care

A critical concept in the school of symbolic interactionism is that there is no social life without communication and shared symbols [1]. There is, in effect, no self. However, by maintaining positive social engagement experiences for end of life PLWD, we avoid many medication interventions through expanded two-way communication opportunities. Families are challenged to learn and identify symbolic and non-verbal communication interactions or representations (such as pictures, touching, hugs, pointing, smiles, gestures and facial expressions etc.). A smile is a smile in any language. Here, family care partners are encouraged to learn to identify the PLWD’s facial expressions as ways of understanding and communicating with them. During end of life care for PLWD, the borders between the self and environment merge so that care partners are challenged to communicate with persons with advanced dementia through the senses. For example, interventions such as arts-based embodiment engagements like the use of elder clowns has been shown to encourage reciprocal communication between PLWD and their care partners [34]. The efficacy of interventions similar to this within the medical model may be limited, but from a sociological perspective they are meaningful.

Person centered care (PCC) seeks to maintain the self throughout the disability [35]. PCC promotes communication in self-determination and empowerment for PLWD, and has been shown to generate better communication outcomes in some situations as opposed to the biomedical approach [36]. At the early stages, PCC can relieve some of the intense stress that accompanies diagnosis for both PLWD and care partners [37]. PCC is a way to connect family care partners to PLWD in highly idiosyncratic ways, drawing from the PLWD’s person centered life history that relates to sensory stimulation. In order to establish PCC with PLWD, care partners can learn how to frame interactions in a manner that generates new meanings within the realm of competencies that PLWD maintain at any given point in their disability [38]. PCC is an alternative to the predominately medical approach to clinical care for PLWD as it attempts to link the person’s lifelong habits and behaviors with their ‘trip back in time.’ 

Activating the five senses (taste, touch, smell, hearing and sight) of the end of life PLWD is a way for family care partners to connect. For example, family care partners have access to the relevant information on the PLWD’s ‘trip back in time.’ Creating photo albums using enlarged photos from the past offers better opportunities for recognition, visual stimulation and non-verbal communication. Family care partners are in a difficult situation because the photos often do not include them but instead the PLWD’s family of origin, parents and siblings. This is an example of how ego can inhibit positive communication. Opportunities abound for family care partners who see the possible benefits of these interventions and aim to help PLWD. 

Verbal communication is difficult when PLWD are bedridden. Namaste is an end of life or late stage dementia care program that utilizes the five senses to cultivate communication with PLWD [39]. The Namaste program is provided 7 days a week and staffed by specially trained persons who provide activities of daily living in a calm manner, with a “loving touch” approach to care [40]. The program takes place in a room with lowered lighting, soft music playing, and the scent of lavender nurturing PLWD to feel comforted, cared for, and cared about in a unique loving environment. Such programs are viewed as vehicles to emotionally connect with bedridden PLWD. By stimulating multiple senses, one can increase the chances of propagating positive interactions and communication. Accessing taste and smell can be achieved through cooking at the bedside using ingredients that produce odors, flavors and sounds from the past. Essential oils that emanate familiar smells and touch using massage, or simply holding hands to convey love and affection, are all legitimate forms of communication. Hearing music from one’s past can evoke positive responses and connections. Other non-verbal forms of communication that are currently used include visual stimuli through Snoezelen (a multi-sensory stimulation therapy in a room created for delivering high levels of stimuli to PLWD), pet therapy, doll therapy, robotics, reminiscence therapy with objects and pictures, and other modifications to the milieu which are impactful [27,39].

## 7. Dementia Citizenship for End of Life Care

Citizenship is a human right that is bestowed on all persons. However, when people are diagnosed with dementia their citizenship is often stripped, similar to that of a prisoner, although they did not choose to have dementia. The concept of dementia citizenship is used in dementia studies to promote the status of discriminated groups of cognitively disabled persons. Dementia citizenship recognizes the self-cognizance of PLWD to exercise rights and responsibilities [41]. PLWD are entitled to live life fully until death. Although the notion of citizenship may not appear to be appropriate for persons with severe dementia in their limited decision-making abilities, it still assumes they want and deserve a full social engagement until death. The citizenship approach is a narrative framework in which a person is included in a community and granted the presumption of autonomy [42]. Research indicates that as a concept it is strongly linked to resilience, an important attribute in end of life care both for PLWD and care partners [30]. Optimizing the social integration and identity of PLWD can have a wide range of positive effects on their course of treatment and quality of life [16]. These outcomes provide incentives for family care partners to stay connected to PLWD.

During the course of time travel for PLWD, it becomes difficult to maintain integration and identity with their present and former self. Identity, in this sense, is inextricably tied to agency that is so important to dementia citizenship. Autonomy, in this sense, is the ability for a person to determine their own life course and agency, and can be defined as a person’s capacity to propel one’s self along that life course. Dementia citizenship is the recognition of those capabilities. It is the treatment that a person receives when they are perceived and interacted with as unique individuals capable of making their own decisions.

The adaptive response to losses associated to those “living with” life-threatening illnesses change as circumstances change. The key is having compassion and genuinely caring for the person. Being with the person living with dementia has the potential to be a spiritual experience where nothing has to be said but hearts connect. The communication occurs on a number of levels. One of the authors found this to be true with his mother (who had dementia) just sitting with her and holding her hand [42]. PLWD go through daily adjustments to their disability. Among the tasks in coping with life-threatening illness the chronic phase is characterized by “living with the disease” [43]. Unlike compressed morbidity, with chronic morbidity the grief process is prolonged. 

Dying is taboo in modernity but even worse is prolonged bedridden experiences. In industrialized countries like the U.S., Japan, or Europe, life-threatening illnesses put everything on pause. In such cultures, the care partner can create difficulties in communication and thwart family support of the PLWD. For example, lack of communication is a common issue among family members of bedridden PLWD [20]. Symbolic interaction and psychological constructivist theories have provided useful understandings of how families create and reconstruct meaning and identity in the midst of loss [44]. These understandings of living with a chronic illness are applicable to dementia [45]. Similar approaches may focus on the ways that meanings frame the process of dying. For example, it is helpful to postulate how individuals and families identify the self or another as “dying” and how these expectations affect subsequent interactions. Such work builds upon early theoretical work in the field [46].

## 8. Discussion

Family members are challenged to eliminate personal egos in dementia care. It is human nature to want to be recognized by PLWD, and to be disappointed when one is no longer known. Yet, with the framework of time travel, there will be a day when we cannot and should not continue reality orientation with PLWD. Social models of care outlined in this paper include the trip back in time; dementia citizenship and sensory engagement modalities for person centered end of life care. In such instances, family care partners play an important role in providing salient symbolic interactions for PLWD and this can add to the quality of life.

In the medical models, elders living at the end of life with dementia can become objectified. Medications are often used to stop PLWD from calling out for help, although such efforts are clear forms of communication to the staff. These residents are lonely and benefit from human contact and social engagement. Family care partners often feel powerless in the absence of training and education to understand how to communicate with their loved one. As change agents, clinical sociologists are interested in empowering families to learn new non-verbal ways to communicate at the end of life. For PLWD end of life care should be tailored to their life history, hobbies, and interests. In the book, *The Veneration of Life*, Diamond writes:

“Alzheimer’s is distinguished from most other diseases in that the ego becomes progressively smaller, allowing more and more of the innate Spirit to become apparent. In contrast, nearly all diseases are characterized by an increased self-concern. In fact, this may very well be at the root cause of the particular disease. As the patient surrenders to the disease, he becomes increasingly more ego-oriented, radiating ever less of his Spirit”.

[42] (p. 4)

The self, or ego, and environment of the bedridden PLWD become one [41]. End of life PLWD have withdrawn into their own social worlds and their vocabularies have shrunk. The environment which surrounds the PLWD becomes their world and their reality. This has immense implications for professional and family care partners who are challenged to use the five senses to effectively communicate with PLWD. End of life therapies have expanded considerably beyond the medical model in the past few years to symbolically link the milieu with PLWD in a powerful dialectic. Treatments that minimize or distract from the depersonalizing experiences that accompany institutionalization include Namaste, Snoezelen and various therapeutic modalities [39,47]. The end of life is full of opportunities to engage in meaningful social connections. The interventions discussed in this paper offer new paradigms for social engagement that can enhance communication. 

## 9. Conclusions

The sociological interventions that deconstruct excessively medicalized dementia care include meaningful social connections for end of life persons with dementia. When family members are empowered by learning how to interact on symbolic levels with PLWD and join them in their time travel, powerful connections can be made. Family dementia education can result in change, while bringing joy to the life of bedridden persons living with dementia.
